# The Role of Immune and Inflammatory Cells in Idiopathic Pulmonary Fibrosis

**DOI:** 10.3389/fmed.2018.00043

**Published:** 2018-03-20

**Authors:** Omkar Desai, Julia Winkler, Maksym Minasyan, Erica L. Herzog

**Affiliations:** ^1^Section of Pulmonary, Critical Care and Sleep Medicine, Department of Internal Medicine, Yale School of Medicine, New Haven, CT, United States

**Keywords:** innate immunity, adaptive immunity, macrophage, lymphocyte, fibroproliferation

## Abstract

The contribution of the immune system to idiopathic pulmonary fibrosis (IPF) remains poorly understood. While most sources agree that IPF does not result from a primary immunopathogenic mechanism, evidence gleaned from animal modeling and human studies suggests that innate and adaptive immune processes can orchestrate existing fibrotic responses. This review will synthesize the available data regarding the complex role of professional immune cells in IPF. The role of innate immune populations such as monocytes, macrophages, myeloid suppressor cells, and innate lymphoid cells will be discussed, as will the activation of these cells *via* pathogen-associated molecular patterns derived from invading or commensural microbes, and danger-associated molecular patterns derived from injured cells and tissues. The contribution of adaptive immune responses driven by T-helper cells and B cells will be reviewed as well. Each form of immune activation will be discussed in the context of its relationship to environmental and genetic factors, disease outcomes, and potential therapies. We conclude with discussion of unanswered questions and opportunities for future study in this area.

## Introduction

Idiopathic pulmonary fibrosis (IPF) is a chronic, progressive, fibrotic disease of unknown etiology characterized by the radiographic and histopathologic pattern of usual interstitial pneumonia (UIP) (1, 2). It is known to have outcomes similar to some cancers, with mortality approaching 50% within 3–5 years after diagnosis (1). Although the origin of this disease is not known, several risk factors have been identified, including cigarette smoking (3), chronic viral infections (4), gastroesophageal reflux (5), and genetic predisposition (6), which will be discussed throughout this article as appropriate. The mechanistic relationship of these risk factors to disease development and progression has yet to be determined.

**Figure 1:**
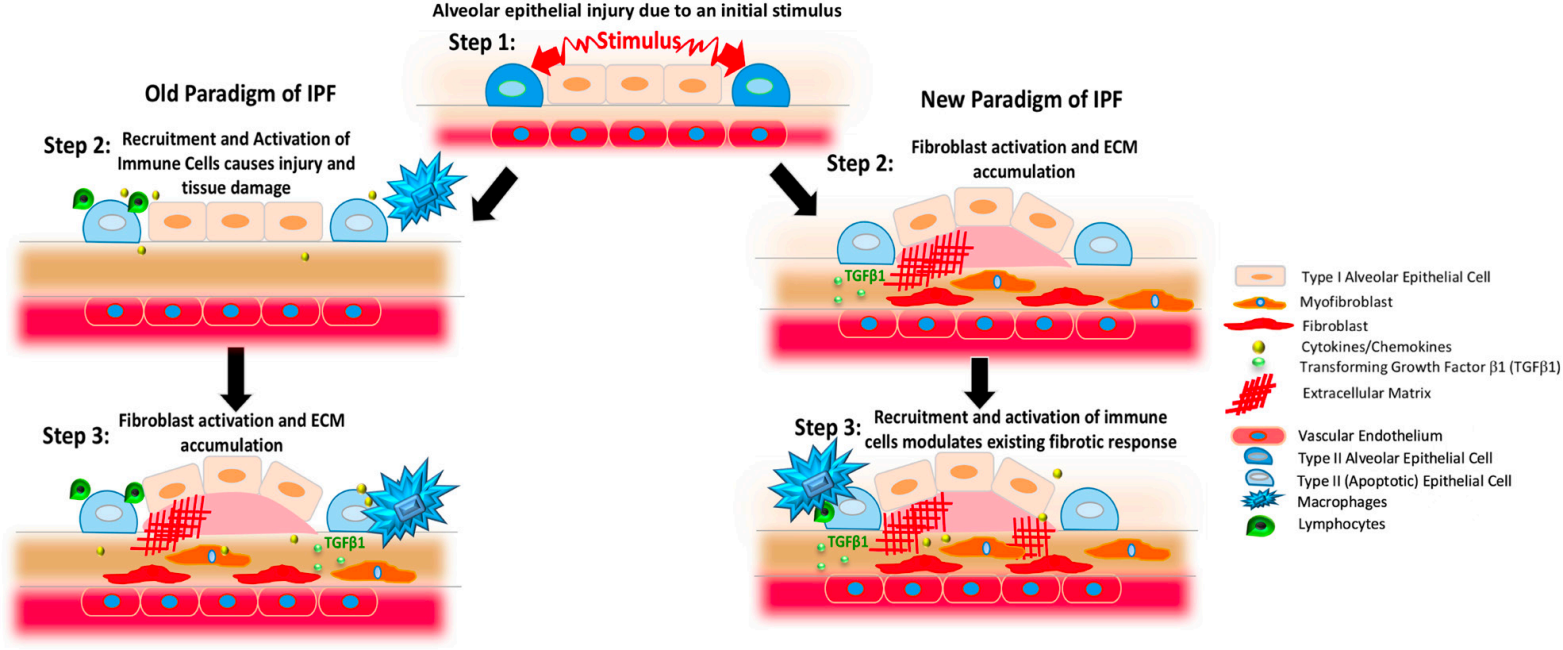
Changing paradigms regarding the proposed pathogenesis of idiopathic pulmonary fibrosis (IPF). The left column presents previous concepts of IPF. In this setting, an initial stimulus affects the alveolar epithelium (blue and pale pink shapes). As shown in the middle panel on the left, this process results in the generation of apoptotic epithelial cells (light blue) and the recruitment and activation of various immune cells including various populations of macrophages (blue) and lymphocytes (green) that produce cytokines and chemokines (yellow). As shown in the third panel on the left, these inflammatory cells and substances induce the activation of fibroblasts (red) and myofibroblasts (orange) to result in TGFβ1 activation (small light green circles) and the deposition of excessively stiff and biochemically abnormal extracellular matrix (ECM, red crosshatched lines). Observations gleaned from clinical trials and experimental modeling have refined this paradigm, however, to result in the scheme shown on the right. In this newer model, the stimuli affecting the lung epithelium leads to fibroblast activation and ECM accumulation that can occur without a primary immunopathogenic component (second panel on left). Once the fibrotic response is established, resident and recruited immune cells, such as macrophages and lymphocytes, modulate existing responses through a variety of mechanisms.

The pathogenic cascade of lung fibrosis is thought to be initiated by perpetuated microinjuries to the alveolar epithelium that engenders a dysregulated wound healing response (7). Through poorly understood processes involving the recruitment and activation of myofibroblasts, normal lung tissue is obliterated by the accumulation of extracellular matrix (ECM) components (8). The basic science and translational research conducted throughout the last few decades has allowed substantial insight into the mechanisms driving IPF (9). In addition, the tireless efforts of investigators conducting clinical trials have resulted in the development of anti-fibrotic therapies with the potential to delay the rate of lung function decline in some patients (10, 11). A central concept of these developments has been the emerging consensus that IPF does not appear to be a direct result of immune cell dysfunction but rather that immune and inflammatory cells can permit, promote, or suppress fibroproliferation driven by native lung fibroblasts (Figure 1). This article reviews the evidence in support of this hypothesis.

## Historical Perspective

In order to understand the controversy surrounding the role of the immune system in IPF a brief overview of the disease state is required. IPF is defined as the presence of UIP in the absence of an identifiable underlying cause. Examination of lung tissue from patients with IPF reveals a paucity, but not absence, of inflammatory cells, when compared to pathologies, such as non-specific interstitial pneumonia, acute respiratory distress syndrome, organizing pneumonia (OP), and granulomatous processes such as hypersensitivity pneumonitis (HP) (12). Similarly, CT scan criteria specify that large areas of ground glass opacities—typically reflective of inflammatory process—are inconsistent with UIP (1). Classical signs of autoimmunity are absent on physical examination and laboratory testing, as is clinical evidence of an identifiable antigen-driven immune response (1). In addition, the long history of failed immunotherapies, such as administration of interferon gamma (13), neutralization of TNFα (14), and suppression of acute inflammatory responses with low dose Prednisone and Azathioprine (15), suggests that IPF does not result from a primary immunopathogenic process. These clinical observations have been interpreted by some sources as indicating the pathogenesis of IPF lacks an immune component (9). However, this array of findings is unlikely to suggest that the immune system is not involved. On the contrary, the worsening of clinical outcomes by classical immunosuppression suggests, if anything, that certain immune responses might be protective and others might be harmful. Thus, better understanding of all forms of immunity has the potential to advance the understanding of IPF.

## Innate vs Adaptive Immunity

The immune response is stratified into innate processes, which respond immediately to chemical or physical patterns of the stimulus, and adaptive immunity, which involves a highly specific antigen-driven response. Both arms of the immune system appear to be activated in IPF. The data supporting this concept are presented below.

## Innate Immune Cells

The innate immune system forms the first line of defense against pathogens. Its recognition of antigens is mainly dependent on pattern recognition by innate immune receptors. These cell populations are central to both host defense and tissue homeostasis. Macrophages and neutrophils are among the best studied innate immune cells in regard to IPF, though a contribution of monocyte-derived cells, such as fibrocytes and myeloid-derived suppressor cells (MDSCs), and of innate lymphoid cells (ILCs), has also been proposed. It should also be noted that parenchymal cells, such as epithelial cells and fibroblasts, also show abnormalities in innate immune activation (16). However, because these stromal populations are not considered to be classical or professional immune cells, their potential and largely speculative contribution to the immunopathogenesis of IPF will not be discussed in this review.

### Macrophages

Macrophages are innate immune cells that not only act as antimicrobial phagocytes in the lungs but also play a key role in the pathogenesis of fibrotic lung disease (17). Of the immunopathogenic mechanisms discussed in this review, macrophage-driven processes are among the most extensively studied with reports of fibrosis-promoting properties dating back nearly 50 years (18). Macrophages can regulate both injury and repair in various models of fibrosis and macrophage heterogeneity has emerged as an important area of study in IPF (9). Prior classification schemes proposed the existing of two phenotypes, namely classically activated M1 macrophages that arise in response to INFγ and TNFα, and alternatively activated M2 macrophages that arise in response to stimulation with IL-4, IL-10, IL-13, and TGFβ1 (19). The central concept has been that M1 macrophages suppress, and M2 macrophages promote, fibroproliferation and uncontrolled repair (17). While recent evidence suggests that a dichotomous stratification oversimplifies the functional heterogeneity of these highly plastic cells (20), the M1/M2 distinction is useful when considering functional distinction in broad terms. In this context, a relative excess of M1 macrophages leads to epithelial cell death and failure of repair such as that seen in acute exacerbation of IPF (AE-IPF), while an excess of M2 macrophages leads to the aberrant and dysregulated repair responses that characterize progressive fibrosis (21). At least one endogenous macrophage-driven pathomechanism identified in AE-IPF is characterized by M2 macrophage activation and upregulation of M2 cytokines (22). Detailed studies performed in several mouse models of IPF demonstrate the heterogeneous and highly plastic nature of lung macrophages, with a contribution from both long-lived resident alveolar macrophages (23), as well as from interstitial macrophages that are at least partially bone marrow derived (24). While the difference in surface marker expression prevents direct translation of highly detailed studies of macrophage subtypes in the mouse, synthesis of the currently available data reveals that the accumulation of cells expressing various scavenger receptors and fibrosis-promoting markers is a common feature of many forms of lung fibrosis including IPF (21, 25, 26).

Macrophages display many functions that frame them as a central contributor to fibrotic responses. As early as the 1980s, alveolar macrophages obtained from patients with IPF were shown to stimulate fibroblast accumulation *via* a paracrine mechanism involving the production of soluble mediators typically associated with alternative activation (18). More recent work using lung-derived macrophages confirms the fibroblast-stimulating properties of macrophages (21), and also reveals that circulating monocytes in patients with IPF appear to be programmed with this property prior to actually entering the lung (21). Further studies using animal modeling reveal that removal (27, 28) or repolarizing (27) of interstitial macrophages is both preventative and therapeutic in several mouse models of IPF. This latter mechanism is the conceptual basis for administration of the large pentraxin protein serum amyloid P to patients with IPF (27, 29), which is currently under investigation for multiple forms of fibrosis including IPF. While several studies indicate that macrophages might also participate in other forms of lung fibrosis *via* the regulation of epithelial cell activation (30), this area remains largely unexplored in the context of IPF.

**Figure 2:**
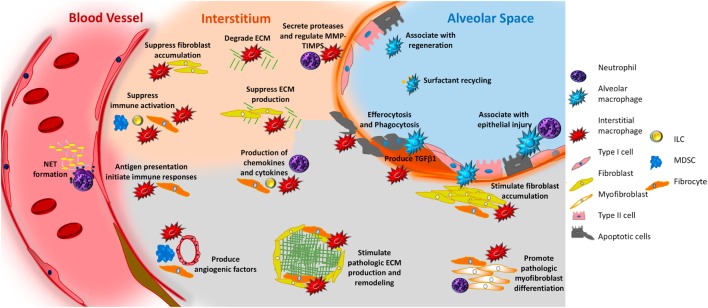
Potential role of innate immunity in pulmonary fibrosis. In response to interactions with pathogen-associated molecular patterns or danger-associated molecular patterns, or to stimulation with various mediators, macrophages—both alveolar (aqua irregular shape) and interstitial (red irregular shape)—can adopt fibrosis modifying properties. These functions include production of TGFβ1, production of soluble mediators that cause fibroblast accumulation and activation, production of TIMPS and MMPS that participate in extracellular matrix (ECM) remodeling, production of angiogenic factors, secretion of lipid mediators, regulation of structural cell injury and stem cell renewal, and surfactant recycling. Neutrophils (purple polymorphonuclear circle) produce neutrophil elastase (NE), TIMPS, and MMPs that dictate whether ECM accumulates or is degraded. Neutrophils also participate in the formation of neutrophil extracellular traps, which may promote fibrosis *via* the production of TGFβ1 and subsequent myofibroblast activation. Circulating fibrocytes (orange spindle shaped cells) are bone marrow-derived mesenchymal cells that enter the lung *via* the vasculature. Once in the lung they adopt multiple functions that would be expected to modulate fibrogenesis including the ability to differentiate into fibroblasts and myofibroblasts, production of ECM, contraction of wounds, participate in antigen presentation, production of chemokines and cytokines, and regulation of angiogenesis *via* production of soluble mediators. Myeloid-derived suppressor cells (MDSC, blue) are immunosuppressive cells that show an association with ECM remodeling and pulmonary hypertension. Innate lymphoid cells (ILCs) produce cytokines that may regulate fibroblast accumulation and ECM production. In the above figure, the functions of each cell are depicted in font matching the cell’s color. Note that cells are not drawn to scale.

A large body of evidence supports the concept that macrophages produce soluble mediators that regulate fibrotic responses (17) but the mechanism(s) through which they adopt this activation state remains incompletely determined. As increasing body of evidence, however, indicates that interactions with dead or dying cells may be involved (31). In this process, called “efferocytosis,” macrophages (either lung resident or recruited) participate in the engulfment of apoptotic cells causes the transcriptional activation of TGFβ (32). In fact, alveolar macrophages produce TGFβ1 in both humans (33) and mice (30) and Cre-mediated removal of TGFβ1 expression in LysM-expressing cells prevents collagen accumulation and histologic evidence of remodeling in several commonly used animal models (30). These results suggest one potential mechanism through which macrophages contribute to fibrosing lung disease. However, in addition to their role in apoptotic cell clearance and TGFβ production, macrophages produce cytokines, such as TNFα, IL-1, IL-6, IL-8, IL-10, and IL-12, and chemokines, such CXCL1, CXCL2, CXCL9, CXCL10, CXCL12, CCL5, CCL17, and CCL18 (34). Their production of lipid mediators such as eicosanoids might contribute to fibrosis (35), though this function has not been specifically studied in either IPF samples or currently used mouse models (36). Macrophages also participate in ECM remodeling through the secretion of matrix metalloproteinases (37) and by direct ingestion and recycling of collagen (38). In other clinical contexts and modeling systems, macrophages are known to direct the metabolic fate of adjacent cells (39), which might carry substantial implications for fibrosis where glycolytic reprogramming has been observed to drive fibroblast activation (40). Macrophages participate in surfactant recycling (41) which could be of critical importance given the known association between mutations in surfactant proteins and susceptibility to IPF (42). Macrophages produce angiogenic factors such as vascular endothelial growth factor (43), which can be both pro- (44) or anti-fibrotic (45), depending upon the timing of expression and the target cell. Macrophages have been shown to rescue intestinal stem cell phenotypes *via* the delivery of WNT-containing exosomes (46) and while a similar effect has yet to be seen in IPF, given the recently reported association between innate immune activation and lung progenitor cell survival (47), it is possible that similar functions may exist in IPF. The potential role of macrophages in pulmonary fibrosis is illustrated in Figure 2.

These data reveal a robust and important relationship between macrophages and fibroproliferation in the IPF lung and lead to the critical question of whether therapies targeting macrophage activation might stabilize or restore lung function in patients with IPF. The few clinical trials conducted in this area have yielded disappointing results. For example, as direct suppression of M1 responses with TNFα neutralization with Etanercept failed to improve clinical outcomes (14). Similar results were seen in a study that sought to inhibit macrophage recruitment *via* treatment with Carlumab, a monoclonal antibody targeting chemokine C-C chemokine ligand 2 (48). While these data could be viewed as negative, they are in fact incredibly helpful as they reveal that targeting the alternative activation state or specific function of macrophages, rather than the M1 phenotype or broadly active aspect such as recruitment, is more likely to be of benefit in a complex disease such as IPF. This concept is the conceptual basis for the ongoing Phase II trial of Pentraxin 5, an acute phase reactant that interferes with innate immune activation by binding to debris and inhibiting Fcγ receptor driven inflammatory process in phagocytic cells (49). The mechanism(s) though which the extrinsic features of the lung microenvironment might result in sterile inflammation and fibrosis are presented below.

## Activation of Professional Immune Cells in IPF: Pathogen-Associated Molecular Patterns (PAMPs) vs Danger-Associated Molecular Patterns (DAMPs)

### Pathogen-Associated Molecular Patterns

A dominant mechanism through which innate immune cells adopt fibrosis-promoting properties likely involves the recognition of innate immune agonists by pattern recognition receptors (PRRs). Ligands for PRRs fall into two classes. Those derived from invading microorganisms are called “PAMPs” and those derived from injured cells and tissues are called “DAMPs” (50). While both inflammatory and parenchymal cells contain PRRs, we will, for the sake of clarity, restrict this particular review to the professional immune cells that have been classically accepted as the first line of host defense (51). Because IPF is not believed to result from a primary infectious process, until recently, the concept of PAMPs has been little studied (31). However, over the last two decades, data gleaned from human and animal studies have linked certain viruses and bacteria with IPF.

### Viruses

Several viruses are proposed as playing a role in the development of IPF. For example, Epstein–Barr virus (EBV, a member of the Herpes family) is enriched in bronchoalveolar lavage (BAL) fluid and lung biopsy tissue of IPF patients when compared with healthy controls (52, 53), and is thought to act *via* upregulating TGFβ1 expression and inducing mesenchymal properties in lung epithelial cells (54). In addition, Cytomegalovirus is thought to accelerate existing fibrosis in bleomycin-treated mice by enhancing TGFβ1 activation and increasing detection of both phospho-SMAD2 and Vimentin (55). A strong association with Herpesvirus saimiri was seen in a human study, wherein the infected epithelial cells demonstrated evidence of IL-17 expression of viral origin (56). Considering the potential contributions of viruses, the use of adjuvant antiviral therapy in IPF has shown potential benefit both in animal models (57) and a small human study (58) although data in this regard are limited.

### Bacteria

The potential contribution of bacteria to IPF pathogenesis is also an area of active investigation. Specifically, a relationship between total bacterial load and poor prognosis was observed in a study in which enriched detection of organisms, such as Haemophilus, Streptococcus, Neisseria and Veillonella, was found in BAL fluid of IPF patients (59). In addition, BAL samples from patients with IPF contain augmented concentrations of certain strains of Staphylococcus and Streptococcus (60), the latter of which was shown in profiling studies to associate with increased NOD receptor signaling and poor outcomes (61). The source(s) of these bacteria is not clear though given the association of IPF with GERD (62), one possibility is that ongoing microaspiration leads to repeated inoculation with oral and gastric organisms. Thus, the concept of the microbiome is gaining traction in IPF and forms the basis for studies examining antibiotics as a novel treatment approach (63).

### Danger-Associated Molecular Patterns

Immune responses are also initiated in the absence of pathogen recognition. Here, damage to previously intact cells and tissue results in the accumulation of substances with the potential to function as “DAMPs.” In uninjured tissues, DAMP-mediated inflammatory processes contribute to homeostasis by allowing the regulated removal of debris, thereby facilitating the resolution of injury and the achievement of repair (31). Abnormal responses to DAMP recognition has been described as one form of immunosenescence (64) and it is intriguing to consider this concept in relationship to the telomerase mutations that are associated with the IPF disease state. Excessive accumulation of DAMPs, however, activates PRRs to engender a microenvironment rich in sterile inflammation (65). These responses may differ from PAMP-driven inflammatory responses (66). Another form of innate immune ligands derived from homeostatic mechanism (HAMPs) has recently been described (67), but because these moieties have not been studied in the context of IPF, they will not be discussed further in our review.

A number of substances are classified as DAMPs. The easiest to conceptualize may be intracellular components such as nucleic acids and organelles that are passively released from necrotic cells. DAMPs might also be actively released by cells *via* the exocytosis of membrane bound vesicles or endosomes. Still another class of DAMPs is generated by the transformation of inert proteins such as collagens into signaling molecules such as collagen fragments. These entities are recognized by innate immune receptors that for the most part have the ability to be activated *via* pathogens as well (31). The activation of these receptors can be protective or harmful depending on the nature of the ligand and the specific receptor. For example, animal modeling reveals that mice lacking toll-like receptor 3 (TLR3) suffer increased fibrosis in the bleomycin model, and the Leu412 Phe polymorphism in the gene encoding TLR3 (which recognizes dsRNA as well as free RNA) has been implicated in a rapidly progressive form of IPF (68). Mice lacking toll-like receptor 4 (TLR4) manifest increased fibrosis in the bleomycin model, and treatment with TLR4 agonists ameliorates fibrosis and remodeling in this setting *via* a mechanism involving lung progenitor cell renewal (47) and augmentation of TGFβ1 and IL-17 production (69). However, because TLR4 inhibition is protective in other settings (70, 71), the role of this PRR is not fully known. A connection between TLR4 and IPF may exist, however, as enrichment of several endogenous ligands for TLR4, such as high mobility group box 1 (72, 73), tenascin-C (74–76), S100 protein (73), and hyaluronan fragments (77), has been reported in the BAL or lung tissue of patients with IPF (78). Furthermore, the finding that a mutation in toll interacting protein, an adaptor protein for toll-like receptor 2 (TLR2) and TLR4, increases susceptibility to IPF (79), suggests a potential protective role for this pathway.

In terms of pathogenic responses, much more information is available. For example, NACHT, LLR, and PYD domains-containing protein 3 (NALP3) inflammasome activation leads to IL-1β production and fibrosis in bleomycin treated animals (80, 81). While the relevance of these results to the human disease state is not directly established, lung tissue from patients with IPF show increased concentrations of uric acid (82), and BAL from these patients contained an increase in free ATP (83), both of which are known inflammasome activators (81, 84). Inflammasome activation in IPF may also occur *via* toll-like receptor 9 (TLR9) as detection of this PRR (85) and its endogenous ligand mitochondrial DNA are both increased in IPF (86). In fact, direct exposure of previously normal fibroblasts to either endogenous TLR9 agonists such as unmethylated CpG-rich DNA derived from mitochondria (mtDNA) (86) or synthetic TLR9 agonists enacts a transition to an αSMA expressing, myofibroblastic phenotype (87). However, because mice with ubiquitous deletion of TLR9 develop worsened fibrosis in several experimental settings (88), likely due to inflammatory nature of these models, the role of TLR9 in IPF has been difficult to understand. Thus, this is an area that would benefit from additional studies and improved models that more accurately represent the microenvironment of the diseased human lung.

### Neutrophils

Neutrophils are innate immune cells that possess several functions through which they might participate in fibrosis. Neutrophilia in BAL fluid has been associated with early mortality in IPF (89) and concentrations of the neutrophil chemoattractant, CXCL8, are increased in IPF (90). Furthermore, levels of alveolar epithelial marker, cytokeratin 19, in BAL fluid correlated to neutrophil concentrations, suggesting an association between neutrophils and epithelial injury in this context (91).

Neutrophils might also contribute to fibrosis *via* their regulation of ECM turnover. Neutrophil elastase (NE), the main proteolytic product of alveolar neutrophils, is increased in BAL fluid of IPF patients (92). NE generates DAMPs by degrading various ECM components, such as collagens I, II, III, IV, fibronectin, laminin, and elastin (93, 94), and *ex vivo* work demonstrates that NE can induce fibroblast proliferation and myofibroblast differentiation (95). NE deficient mice are protected from the fibrosis seen in both the bleomycin and asbestos models (93, 95), and the NE inhibitor, Sivelestat, is protective in the bleomycin model (96). Neutrophils also control ECM homeostasis through their regulation of the net balance between MMPs and TIMPs (97, 98), particularly the pro-fibrotic MMP-2, MMP-8, and MMP-9 (99). Batimastat, a synthetic inhibitor of MMP, when used in bleomycin-induced mice, resulted in reduced MMP-2, MMP-9, and TIMP-1 level in BAL fluid and was, therefore, useful in preventing pulmonary fibrosis (100), though the relevance of these findings to human IPF remains unclear.

One newly described fibrosis-promoting function of neutrophils is the generation of extracellular neutrophil traps. These pro-inflammatory collections of chromatin and neutrophils regulate both immune cell function (101) and fibroblast activation (102). While enhanced detection of intrapulmonary neutrophil extracellular traps (NETs) has been reported in both the bleomycin model and in some forms of fibrotic ILD (102), a specific association with IPF has yet to be fully described. Further studies are warranted to understand whether NETS play a role in IPF pathogenesis.

To summarize, neutrophils are innate immune cells that are associated with the production of cytokines and chemokines, presence of injury, regulation of ECM turnover, and generation of NETs. All of these functions would be expected to result in fibroblast activation and ECM accumulation (Figure 2). However, because the pathology of UIP is not characterized by neutrophil accumulation—and, in fact, the presence of neutrophils would lead to a pathologic diagnosis other than UIP—the role of neutrophils remains unclear.

### Fibrocytes

Fibrocytes are circulating, bone marrow-derived mesenchymal progenitor cells that can migrate into lung tissue and further differentiate into fibroblasts and myofibroblasts (103). These cells are believed to derive from monocyte-based progenitors. They comprise only a small fraction of circulating leukocytes in normal humans, but are found abundantly in pathologic conditions characterized by macrophage-driven inflammation and persistent fibroblast activation such as IPF (104). These cells express hematopoietic and progenitor cell markers, CD45 and CD34, and also produce ECM proteins such as collagens I and III, vimentin and fibronectin (105). Similarly, they can be induced to express αSMA (106) and participate in the contraction of artificial wounds (107, 108). However, despite their documented ability to both produce ECM and display functions of myofibroblasts in a variety of modeling systems, consistent evidence of these properties in human lung tissue remains scarce. Thus, in recent years, attention has focused on alternate functions of these highly plastic cells. Fibrocytes not only express receptors for chemokines, such as CCR3, CCR5, CCR7, and CXCR4, but also produce inflammatory cytokines, TNF-α, IL-6, IL-8, and IL-10, and chemokines, MIP-1α, MIP-1β, MCP-1, and GRO-α (109, 110). They participate in antigen presentation (111, 112) and angiogenesis (113), and in some settings are able to control the activation of adjacent fibroblasts *via* paracrine means (114). Thus, fibrocytes display an array of functions that would be expected to influence the development and progression of lung fibrosis (Figure 2), though their specific contribution to IPF is currently not defined and requires further investigation.

### Myeloid-Derived Suppressor Cells (MDSCs)

Myeloid-derived suppressor cells are a heterogeneous group of immature myeloid immune cells, which appear to be related to poor prognosis in certain forms of cancers (115). MDSCs play a role in the immune system through their suppressive action on T cells (116). They promote regulatory T cell (Treg) expansion and restrain T-cell activation (117) and are associated with a number of diseases characterized by fibrosis and pathologic remodeling. Thus, it is perhaps not surprising that one recent study found that elevated concentrations of peripheral blood MDSCs, defined by the surface markers HLA-DR, CD33, CD11b, CD14, and CD66b, reflect poor lung function in patients with IPF (118). Another experiment with bleomycin-induced mice showed that MDSCs triggered vascular remodeling and pulmonary hypertension, and that preventing their recruitment *via* neutralization of CXCR2 normalized the pulmonary pressure (119). While the relationship between vascular abnormalities and parenchymal fibrosis remains poorly understood, accumulating evidence suggests that these events might significantly impact parenchymal homeostasis during injury and repair (45). Thus, therapeutic strategies targeting the activity of MDSCs or restricting their accumulation and expansion in peripheral blood may be a novel approach to disease modification, though more work is needed to understand their relevance to human disease (118).

### Innate Lymphoid Cells

Innate lymphoid cells are newly identified lymphoid cell populations that do not express the recombination activating gene and are classified into three subgroups: ILC1, which include natural killer cells that produce IFN-γ as well as CD127lo and CD127hi ILCs (120); ILC2, which produce type 2 cytokines, such as IL-5 and IL-13; and lastly, ILC3 that produce IL-17 and IL-22 (121). ILCs in the lung interact with epithelial cells, natural killer T cells, and myeloid cells to form an immune system network (122). ILC2 are activated quickly by environmental antigens and pathogens to release large quantities of IL-13, thereby suggesting a potential role in pulmonary fibrosis (123). ILCs have been identified in the IPF lung (124) though studies of their role in this disease remain in the nascent stages. Thus, this is an area that would benefit from additional study.

## Adaptive Immune Responses: T Cells and B Cells

As noted above, the role of lymphocytes in fibrosis is poorly understood and controversial. The failure of IPF to improve in response to lymphocyte-modulating therapies, and the observation that lymphocytes are not required for the development of experimentally induced fibrosis in mice (125), has contributed to this situation. However, substantial and outcome predictive abnormalities in lymphocyte subsets and activation have been described in the lungs and blood of IPF patients, and animal modeling shows that certain lymphocyte populations are sufficient to induce or modify mammalian lung fibrosis. Thus, lymphocytes might participate in fibrosis *via* as yet undefined mechanisms. The evidence supporting this concept is reviewed below.

### T Lymphocytes

**Figure 3:**
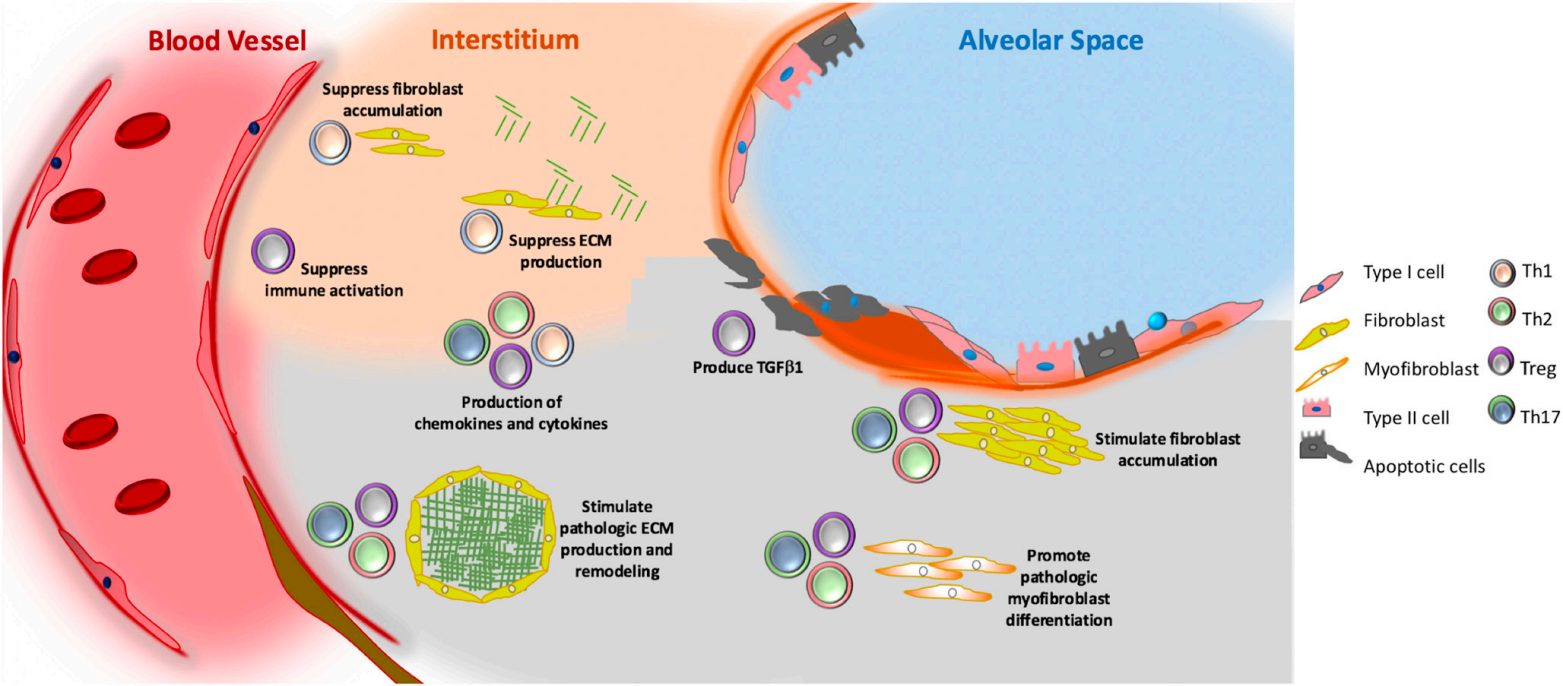
Putative role of adaptive immunity in idiopathic pulmonary fibrosis. Th1 (grey) cells may suppress fibroblast responses through the secretion of pro-inflammatory cytokines, such as interferon gamma and TNFα. Th2 (pink) and Th17 (green) cells stimulate fibroblast proliferation, activation, and extracellular matrix (ECM) production through their secretion of IL-4 and IL-13 (Th2) and IL-17 (Th17). Tregs (purple) may either promote fibrosis through production of PDGFβ and TGFβ1, or suppress fibrosis *via* poorly understood effects on fibrocyte accumulation. Note that image is not drawn to scale.

Relative to samples obtained from normal individuals, lung tissue and BAL fluid from patients with IPF are enriched for several population of T lymphocytes (126). In the tissue, these lymphoid aggregates contain CD3+ T lymphocytes (127) and evaluation of the peripheral blood supports these findings to some extent. Specifically, transcriptional profiling of PBMCs found that a signature characterized by reduced expression of T cell regulatory genes related to the immune checkpoint CTLA-4 was associated with reduced event free survival (128) and these findings were recently recapitulated in a landmark study involving six independent IPF cohorts from centers across the US and Europe (129). These findings recapitulate an earlier study in which reduced expression of the costimulatory molecule CD28 on circulating T cells was seen to be a predictor of poor outcomes (130). Because treatment with the lymphocyte-modulating agent Azathioprine results in impaired function of molecules that function as immune checkpoints (131), it is intriguing to speculate that the worsened outcomes seen in the Azathioprine-treated subjects in the PANTHER trial (15) relates to aberrant activation of CD4+ T lymphocytes. In this light, it is also interesting that the checkpoint inhibitors used as cancer immunotherapy can cause inflammatory ILD (132) though to date a specific relationship with IPF has not been shown. CD4+ T lymphocytes are divided into several subpopulations, among which the best studied in IPF are T-helper cells, as shown in Figure 3. T-helper cells comprise several classically defined subpopulations based on their pattern of cytokine expression. In the below paragraphs, the data regarding specific T-helper populations in the context of IPF will be presented.

### Th1/Th2 Cells

Perhaps the most studied concept in T-helper biology as it relates to pulmonary fibrosis is the contributions of Th1 and Th2 cells. Animal modeling, and some human data, suggests that the relative proportions of these populations might enact the balance between injury and repair. Here, Th1 cells and their secretory products are thought of as being anti-fibrotic and Th2 cells and their mediators are considered pro-fibrotic (133). For example, Th1 cells release IL-12, which is a potent inducer of IFNγ, and several studies have reported a relative reduction in IFNγ levels in the BAL or circulation of patients with IPF (134). In addition, a bleomycin study in mice found that IL-12-attenuated pulmonary fibrosis *via* modulation of IFNγ production, thus presenting a protective role of Th1 associated mediators in fibrosis (135). These findings are supported by other work showing that attenuation of Th1 differentiation *via* targeting of the transcription factor T-bet increased bleomycin-induced lung injury (136). Conversely, studies that focused on the Th2 cytokines IL-4 and IL-13 showed them to stimulate fibroblast proliferation, collagen production, and fibroblast to myofibroblast differentiation—thereby rendering Th2 cells fibrogenic (137). In addition, detection of Th2 cells and their secretory mediators appears to be enhanced in the lungs and blood of patients with IPF (137–140). However, systemic administration of recombinant IFNγ (which would simulate the presence of Th1 biology) and monoclonal antibody-mediated neutralization of IL-13 (which would specifically target Th2 responses) failed to demonstrate efficacy in randomized, placebo-controlled trials of patients with IPF (141, 142). Therefore, the concept of the Th1/Th2 balance as a central mediator of IPF may require re-evaluation and the development of strategies to better and more efficaciously target their secretory products. The potential role of Th1 and Th2 cells is shown in Figure 3.

### Th17 Cells

The role of Th17 cells in IPF is also incompletely defined. Th17 cells produce cytokines, such as IL-17 and IL-22, which are host-defensive cytokine in many infectious conditions but also promote inflammatory pathology in various diseases such as autoimmune conditions (143). As shown in Figure 3, the functions of IL-17 include stimulation of ECM production, collagen disposition, and mediation of TGF-β signaling (144). Increased detection of IL-17 in the lung tissue, BAL, and serum of IPF patients suggests a potential relationship with disease. These human findings are supported by murine studies in which administration of IL-17A is sufficient to induce collagen accumulation and fibrotic lesions (144, 145), and that neutralization of IL-17 can reduce fibrosis in several animal models (146, 147). Furthermore, in silica-induced lung fibrosis, neutralization of IL-17A delayed T-cell-driven immune responses and consequently slowed the progression of lung inflammation and fibrosis (148). Interestingly, recent work has expanded the concept of IL-17 in fibrosis beyond lymphocytes, as one recent studying in an experimental model of HP found neutrophils and monocytes/macrophages to be a dominant source of IL-17A (149). Similar findings have not been described in IPF. While IL-22, another product of Th17 cells, appears to be protective in the bleomycin model (150) BAL concentrations do not differ between IPF and control (151). Because anti-IL-17 treatment has not been tested in IPF, the efficacy of neutralizing Th17 cells and their secretory products as a therapeutic approach in IPF is currently not known.

### Regulatory T Cells

The role of Tregs in pulmonary fibrosis has been gaining acceptance in the recent years. Due to their ability to produce both IL-10 and TGFβ1, Tregs have the potential to both promote or suppress fibrosis depending on the context. For example, a now seminal 2009 study reported marked suppression of functional CD4+, CD25high, FoxP3+ cells in the BAL and peripheral blood of IPF patients (152), thereby showing, for the first time, a relationship between impaired Tregs and IPF. However, more recent studies in this area have actually shown an increase in Tregs, and an imbalance of the Treg/Th17 axis in IPF patients (153). In addition, a population of aberrantly activated Tregs identified by expression of the neuroimmune molecule Semaphorin 7a+ was sufficient to engender TGF-β1 induced fibrosis in the adult mouse lung (154) *via* undefined mechanisms. A synthesis of this information suggests that recruited or lung resident Tregs might be fibrosis-suppressive or fibrosis-stimulatory depending on their interaction with the local milieu (155). This concept is supported by experimental data from a bleomycin model showing that Tregs may stimulate TGFβ1 production and collagen accumulation when present during the injury phase, but might reduce these endpoints when present during the later stages of the model (155). These studies are complemented by data from an LPS model of lung injury showing that Tregs suppress fibrocyte recruitment and fibrosis *via* interruption of the chemokine C-X-C motif ligand 4/stromal cell-derived factor 1 (CXCL4/SDF1) axis (156), but promote fibroblast activation *via* production of PDGFβ in a model of silicosis (157). In summary, Tregs play a controversial role in pulmonary fibrosis and depending on the stage of fibrosis, they can be both harmful as well as protective. The putative contribution of Tregs to mammalian lung fibrosis is depicted in Figure 3.

### B Cells

B cells represent another arm of the adaptive immune system. Functioning primarily as producers of antibodies, increased detection of CD20+ B cells has been reported for IPF lungs (158). A variety of novel autoantibodies targeting neoepitopes have been reported in IPF (159–163), with many of the targets being structural cell proteins, such as desmoplakin (164) and periplakin (159). In addition, while the presence of clinically relevant positive serology effectively rules out IPF as a diagnosis, recent work demonstrates that patients with low level titers of autoantibodies might have worsened clinical outcomes than those patients lacking these findings (160). Presence of autoantibodies can be linked to poor survival as seen in a recent study wherein high levels of anti-vimentin were associated with worsened pulmonary function and prognosis (165). Similar findings were observed with the presence of anti-HSP70 autoantibodies in patients with IPF (166). Further evidence of a potential role for humoral responses in the pathogenesis of IPF is provided by the detection of antibodies targeting BPI fold containing family B, member 1 (167) though to date the mechanistic impact of these observations remain elusive. While B cell subtypes and function have not been specifically phenotyped in large-scale clinical studies, evidence does exist supporting a role for B cells in some forms of this disease. For example, detection of B lymphocyte stimulator, which is also known as B-cell-activating factor (BAFF), is enriched in the lungs and blood of patients with IPF (168). The potential of B cells to serve a mechanistic role in this disease is shown by a retrospective study in which stable outpatients with end stage ILD who received CD20 targeted therapy (which removes B cells) showed a trend toward improved lung function (169). A beneficial role of B-cell-targeted therapies is further supported by the finding that patients with respiratory failure due to an AE-IPF showed clinical improvement after undergoing combined plasmapheresis (which would be presumed to remove offending antibodies) and anti-CD20 therapy (170). These human observations are complemented by animal modeling in which neutralization or genetic ablation of BAFF attenuates pulmonary fibrosis (168). However, because data gleaned in other models show that B cells may actually suppress fibrotic response (171), precise understanding of the relationship between B cells and IPF remains elusive and the potential contribution these cells to IPF pathogenesis remains a query in need of further study. Due to the largely speculative nature of their relationship with fibrosis, B cells are not included in Figure 3.

## Conclusion

**Figure 4:**
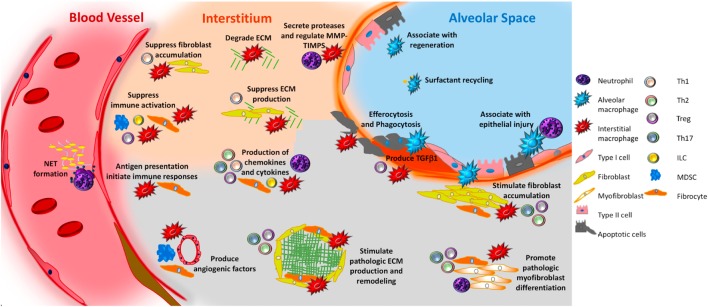
Unifying schematic of immunopathogenic mechanisms of idiopathic pulmonary fibrosis (IPF) reveals that many important fibrosis-promoting processes may be regulated by input from both the innate and adaptive immune systems. Currently available data suggest that innate mechanisms may dominate. For example, both alveolar and interstitial macrophages respond to innate immune ligands present in pathogen-associated molecular patterns or danger-associated molecular patterns to assume adopt fibrosis modifying properties, including production of TGFβ1, paracrine regulation of fibroblast accumulation and activation, production of TIMPS and MMPS that participate in extracellular matrix (ECM) remodeling, production of angiogenic factors, secretion of lipid mediators, regulation of structural cell injury and stem cell renewal, and surfactant recycling. Macrophages might also suppress fibrosis by stimulating a regenerative program in epithelial stem cells, by regulating MMPs and TIMPS, and by directly degrading collagen and ECM. Neutrophils may promote fibrosis *via* the formation of neutrophil extracellular traps (NETs), which may promote fibrosis *via* the production of TGFβ1 and subsequent myofibroblast activation. However, neutrophils may also suppress fibrosis by regulating the MMP:TIMP balance and producing neutrophil elastase (NE) which degrades ECM. Circulating fibrocytes possess several fibromodulatory, including the ability to differentiate into fibroblasts and myofibroblasts, production of ECM, contraction of wounds, participate in antigen presentation, production of chemokines and cytokines, and regulation of angiogenesis *via* production of soluble mediators. Myeloid-derived suppressor cells (blue) display show an association with ECM remodeling and pulmonary hypertension. Innate lymphoid cells (ILCs) produce cytokines that may regulate fibroblast accumulation and ECM production. In terms of the adaptive immune response, Th1 cells may suppress fibroblast responses through the secretion of pro-inflammatory cytokines while Th2 and Th17 cells stimulate fibroblast proliferation, activation, and ECM production. Tregs either promote fibrosis through production of PDGFβ and TGFβ1, or suppress fibrosis *via* poorly understood effects on fibrocyte accumulation. B cells are not shown in this figure given the largely speculative nature of their role in IPF. The redundancy and opposing effects of these functions likely accounts for the failure of IPF to respond to classical forms of immunosuppression. Given the pronounced contribution of the innate immune system, interventions targeting the recognition of, or response to, innate immune ligands might be of benefit.

As can be seen, the cells of the immune system show a rich and multifaceted contribution to IPF. When viewed in contrast with the epithelial cells that are believed to be the primary site of injury, and with fibroblasts, which are viewed as the driver of matrix deposition and remodeling, the more heterogeneous contribution of the innate and adaptive immune systems shown in Figure 4 is nuanced and unlikely to respond to a single intervention. This aspect, combined with the relative ease of isolating immune cells and substances from the BAL and circulation renders the immune system an attractive area for the development of immunopathogenesis-based personalized therapies based on easily accessible biomarkers. Areas of particular interest and important questions in this context, which are shown in Box 1, would benefit from concerted efforts performed in large-scale multicenter recruitment efforts, leveraging of existing datasets and registries, and the generation of improved modeling systems that more faithfully recapitulate the complex microenvironment of the fibrotic human lung and improve the understanding and treatment of IPF on a global scale.

Box 1 **Unanswered questions regarding the immune and inflammatory cells in idiopathic pulmonary fibrosis (IPF).**

- To what extent do data obtained from mouse models reflect the situation in the fibrotic human lung? Can mimetics be developed that more accurately simulate the IPF disease state?
- Do events in the peripheral blood truly reflect events occurring in the diseased lung?
- Do the innate immune abnormalities seen in IPF represent a unique form of immunosenescence?
- Can therapies targeting macrophage activation stabilize or restore lung function in patients with IPF?
- Does the altered microbiome cause pathogen-associated molecular pattern-driven innate immune activation in IPF and are antimicrobial therapies efficacious in IPF?
- Does perpetuated microinjury cause danger-associated molecular pattern (DAMP)-driven innate immune activation in IPF and are therapies targeting DAMPs and their receptors efficacious in IPF?
- Are neutrophil extracellular traps an important part of IPF pathogenesis?
- What is the role of fibrocytes and myeloid-derived suppressor cells in IPF?
- Do innate lymphoid cells participate in IPF?
- How does the relative balance of T-helper cells participate in IPF and can this contribution be targeted in a safe and efficacious manner?
- Are B cells involved in the development of IPF?
- Can immune events detected in the circulation be used to guide personalized therapies in IPF?

## Author Contributions

OD wrote manuscript, JW made figures and wrote manuscript, MM wrote manuscript, and EH wrote manuscript. All authors approved final version.

## Conflict of Interest Statement

The authors declare that the research was conducted in the absence of any commercial or financial relationships that could be construed as a potential conflict of interest.
